# Newborn screening: the genomic challenge

**DOI:** 10.1002/mgg3.74

**Published:** 2014-03-13

**Authors:** Harvey L Levy

**Affiliations:** 1 Division of Genetics and Genomics, Boston Children's HospitalBoston, Massachusetts; 2 Department of Pediatrics, Harvard Medical SchoolBoston, Massachusetts

We have just celebrated the 50th anniversary of mandatory newborn screening (NBS). Beyond any question, NBS has been a huge success. It has virtually eliminated the tragedy of intellectual disability from phenylketonuria (PKU) and congenital hypothyroidism in the developed world. It has eliminated death or profound neurologic sequelae from neonatal sepsis in galactosemia and in salt-wasting congenital adrenal hyperplasia and sickle cell disease. The clinical complications of biotinidase deficiency are now rarely encountered. Sudden death from the fatty acid oxidation disorders is almost a thing of the past. Children with homocystinuria and maple syrup urine disease can achieve their full potentials and grow into productive adults.

If this can be accomplished for the metabolic and endocrine disorders, could there be even greater benefit from NBS for genetic disorders in general, including nonmetabolic genetic disorders? True, we do not have preventive therapies for chromosomal aberrations or most other genetic abnormalities but there could be many other benefits from neonatal diagnosis, such as information for the family to prepare for progressive disability in the child, for genetic counseling for family planning, for prenatal or preconceptual diagnosis in future pregnancies, for prevention of needless and expensive diagnostic odysseys in the child, and still other potential benefits (Landau et al. 2014).

So, why not expand NBS into genetic screening? This probably can be done. Next-generation sequencing (NGS) could allow examination of the entire genome. The cost of the sequencing is rapidly decreasing and may soon be low enough to accommodate NBS. Potentially, every genetic alteration could be identified within a few days after birth by testing the current NBS specimen or perhaps even earlier by screening umbilical cord blood. If screening included identifications of variations considered to increased risk for common diseases such as cancer, Alzheimer's disease, or Parkinson's, the infant would also be a proxy for family members leading to their testing for at-risk variations. Newborn genetic screening sounds like a “no-brainer.”

## Unintended Consequences of Current NBS

While it is true that NBS has led to significant benefit in the diagnosis and treatment of many disease, it is also true that current NBS has serious unintended consequences (Wilcken 2013). From its very beginning, as a method that led to presymptomatic diagnosis and preventive treatment for one disorder, PKU, overdiagnosis, and overtreatment has been an issue (Paul and Brosco 2013). For instance, when NBS for PKU began, it was assumed that every infant with an elevated phenylalanine level had PKU and required dietary therapy. Within a few years, however, this was shown not to be true; some of these infants had a variant of PKU characterized by a lower level of hyperphenylalanemia and did not require therapy (Kennedy et al. 1967). As NBS evolved overdiagnosis and unnecessary treatment has substantially increased, especially so with the relatively recent addition of expanded NBS by tandem mass spectrometry (MS/MS). Instead of this happening in one or two disorders, it is now occurring in many disorders (Wilcken 2013).

As an example, medium chain acyl-CoA dehydrogenase deficiency (MCADD) has been known to result in sudden death. Consequently, every infant found by NBS to have any increase in octanoylcarnitine (C8), the major biochemical marker for MCADD, is assumed to be at risk for sudden death. However, the available C8 levels in children with MCADD who suddenly died have always been markedly elevated, suggesting that the risk of sudden death may only apply to infants in whom the C8 level in NBS is unusually high (Yusupov et al. 2010). Nevertheless, attention is called to infants found by NBS to have even very low elevations of C8 and many are “medicalized” by metabolic follow-up and treatment. In our center we are seeing more cases of several other metabolic disorders since expanded NBS began than came to clinical attention prior to NBS. NBS for galactosemia is another example. Most infants with a positive NBS result have a benign variant of galactosemia known as the Duarte/galactosemia genetic compound (Levy et al. 1978; Ficicioglu et al. 2008). Nevertheless, these infants are usually referred to metabolic centers where expensive confirmatory testing may be performed and some unnecessarily treated with a lactose-free diet (Ficicioglu et al. 2008). The story of benign variants identified in NBS continues through very long chain acyl-CoA dehydrogenase deficiency (VLCADD), isovaleric acidemia (IVA), congenital hypothyroidism, and most likely every disorder screened (Vockley and Ensenauer 2006; Ensenauer et al. 2004).

Beyond these likely benign variants, several entire disorders identified in NBS are likely benign. These disorders are among the most frequently identified in NBS and include short chain acyl-CoA dehydrogenase deficiency (SCADD), a fatty acid oxidation disorder (Waisbren et al. 2008), methionine adenosyltransferase (MATI/III) deficiency (Mudd et al. 1995), isobutyryl-CoA dehydrogenase deficiency (12), and 3-methylcrotonyl-CoA carboxylase deficiency (3-MCCD) (Landau and Levy, unpubl. data). Infants with the NBS abnormality suggesting these disorders, however, are usually referred to a metabolic center where confirmatory testing is performed and are often followed up for several years with medical visits and laboratory tests as well as treatment.

None of these unintended consequences are reasons to conclude that NBS has been a net negative. As mentioned at the outset of this commentary, NBS has been an extraordinarily positive force in the prevention of tragedy from many disorders. They are reasons, however, to be very cautious in expanding NBS into “genetic screening.” Even with caution we can be certain that there will be unintended consequences. Without careful and intelligent planning, the consequences could be extremely disruptive to many families. With informed planning, the negative consequences could be minimized so that newborn genetic screening could be a very positive force in medicine.

## What is Meant by Genetic NBS?

The current blood specimen collected from the heel of the newborn infant or blood collected from the umbilical cord would be tested by whole exome sequencing (WES) or whole genome sequencing (WGS), presumably within current NBS programs. Should some NBS laboratories not be capable of this highly sophisticated testing, as is very likely, the NBS program could contract out genetic screening to medical centers or private laboratories with this capability. Genetic variations considered to have clinical significance would be reported to the medical care provider or to a genetic center leading to evaluation of the infant. Many more genetic disorders than the current 25–30 metabolic disorders would be covered. These would include additional metabolic disorders such as the Smith–Lemli–Opitz (SLO) syndrome, the congenital disorders of glycosylation (CDG), and others. Genetic screening would also expand NBS into nonmetabolic genetic disorders such as chromosomal abnormalities, neurofibromatosis, Duchenne muscular dystrophy, tuberous sclerosis, and many others (Table 1). The clinical phenotype might be apparent at birth or within the first weeks or months of life, or maybe later in onset, appearing in childhood or the adult years. Treatment might consist of a specific therapy that could benefit the infant or information could be provided to the family about the potential for recurrence that might influence future family planning. Genetic variations that indicate a certain degree of risk for common diseases such as cancer, diabetes, cardiomyopathy, or Parkinsonism might also be reported leading to genetic and clinical evaluation of family members.

**Table 1:** A brief sample of the additional genetic disorders potentially identifiable by genomic sequencing in newborn screening.

Disorder	Treatment	Other potential benefit
Smith–Lemli–Opitz syndrome	Cholesterol	Family planning^1^
Duchenne muscular dystrophy	Drug (?)	Patient planning
		Family planning
Congenital disorders of glycosylation	None^2^	Family planning
Neurofibromatosis	None	Patient planning
		Family planning
Wilson disease	Penicillamine	Family planning
Menkes disease	Copper	Family planning
Lysosomal storage disorders	Enzyme	Family planning

1 Family planning includes prenatal or pre-implantation diagnosis.

2 The exception is type 1b in which mannose therapy may be very effective.

The extent to which genetic variations might be reported would have to be determined prior to the initiation of genomic sequencing. One plan could be to examine only those areas of the genome in which there could be variations considered “actionable.” The “actions” could include therapy or diagnosis so as to avoid medical odysseys or information upon which the family may act (Berg et al. 2011). The remaining sequences could be masked or variations in them could be identified but not reported. This latter information could be deleted or could be stored for future recovery should there be a clinical need. Another plan could “open up” the sequencing so that all variations believed to possibly be of clinical significance would be reported. This would include mutations known to be pathological as well as variations in which the evidence for disease association is equivocal. This plan would also include all variations that are believed to indicate a higher than average risk for late onset common diseases as well as many others.

## The Potential for Unintended Consequences in Genetic NBS

Should NBS be expanded into genetic screening? It is certain that unintended consequences will occur, some likely to be quite serious. The sequencing is unlikely to be totally reliable and errors will almost certainly occur (Zhu and Xiong 2012). Depending on the sequencing platform used, some variations will be missed (Clark et al. 2011), potentially depriving an infant of early diagnosis and ameliorative or preventive therapy. Interpretation will vary among screening laboratories, one program assigning a variation as a pathological mutation and another program considering the variation inconsequential. This will derive from the uncertainty of many genetic variants as causal of disease, an uncertainty that will not only continue but likely increase as routine genetic screening identifies a great many more variants than are now known (Cooper and Shendure 2011). This inconsistency in assigning causation of disease to genetic variants will likely result in both underdiagnosis and overdiagnosis of disease. Even when there is certainty that a variant is associated with a known disorder the consequences of the variant, within the disorder or even the consequences of the disorder itself, may be unclear. This has plagued the recent expansion of metabolic disorders in NBS and will surely be increased by genetic NBS (Clayton 2010; Wilcken 2012).

A major concern is the possibility that some parents might opt out of NBS entirely from their general opposition to DNA examination or fear that the detection of genetic variations in their newborn will jeopardize obtaining health insurance or life insurance, or even school acceptance and future employment (Landau et al. 2014). This would have tragic consequences for an infant with PKU or congenital hypothyroidism or any other disorder in which presymptomatic therapy would prevent the abnormal phenotype. Very few parents opt out of current NBS but the likely requirement of informed consent for genetic screening could threaten universal NBS.

## Challenges of Incorporating Genetic Screening into NBS

Could genetic screening become incorporated into the NBS of today? What are the implications for medical and clinical genetic follow-up of genetic NBS? It is unlikely that the current public health-related NBS laboratories, with very few possible exceptions, will be able to perform and interpret NGS. Consequently, a new structure for NBS will be required, likely a two-tiered approach in which the current NBS laboratories would continue to perform the mandated NBS and other laboratories would perform genetic NBS. This will then require either sharing of the NBS specimen or a second NBS specimen which will be either cord blood collected on filter paper or another blood collected from the heel of the infant. Genetic NBS will almost certainly require informed consent. Who would provide the complicated genetic information to the parents that truly informed consent will require? Will this be the responsibility of the nurses or the attending physicians? If so, will they have the time and knowledge sufficient for this purpose? Will genetic counselors perform this service? If so, they would only be available at medical centers and not at the many more smaller hospitals where most babies are born. Would a descriptive brochure be sufficient for informed consent? If so, would most parents have the time and the background to understand the genetic information? At the very least, genetic NBS would impose major new obligations upon NBS and on the hospitals.

Follow up of NBS findings is a major requirement of NBS (AAP Newborn Screening Task Force 2000). Infants with significant findings in current NBS are usually evaluated at medical centers with confirmatory testing. This requires biochemical analyses and may also require genotyping (Landau et al. 2014). Findings in genetic NBS would likely require medical genetic evaluation with confirmatory sequencing as well as additional testing. Given the many newborn infants who could be referred for genetic evaluation, this could overwhelm the existing complement of medical geneticists and significantly increase the already very high costs of medical care.

## Inevitability of Genetic NBS

Despite all of the potential difficulties, genomic sequencing to one degree or another will eventually be incorporated into NBS. Studies show that parents are interested in genetic screening of their newborns (Goldenberg and Sharp 2012). Before incorporation into universal NBS genetic screening will likely be limited to those relatively few areas of the genome wherein genes associated with known actionable disorders reside. Incidental findings and at-risk variations would not be reported. It will also likely begin by being offered as a supplement to mandated NBS only in medical centers with the complement of staff that can provide the appropriate information required for informed consent and in areas where medical genetic follow-up can be obtained. Genetic screening will likely be considered additional or supplemental NBS and the family that consents will be assessed an additional charge for neonatal care since neither insurance nor the hospitals are likely to cover it, although some hospitals might offer this without additional charge as an inducement for families to deliver there. Medical centers and free-standing private genetic laboratories will compete for this service and many private laboratories will solicit online for this additional NBS.

Any genetic NBS raises many questions of feasibility, effectiveness, and, certainly not least, ethics and the medico-legal. The National Institute of Child Health and Development (NICHD) is currently funding four 5-year research projects to examine the application of NGS to NBS (http://www.nih.gov/news/health/sep2013/nhgri-04.htm, accessed 29 January 2014). Each of these projects is to examine technical feasibility in applying genomic sequencing to NBS, to test the medical effectiveness of sequencing in a neonatal setting, and to address the ethical, legal and societal implications of sequencing in NBS. One would hope that genetic sequencing in the newborn would wait until these projects have been completed and the results become known.
